# Interdisciplinary Management of Biological and Prosthetic Implant Complications in the Esthetic Zone: A Case Report

**DOI:** 10.1155/crid/7397624

**Published:** 2025-09-04

**Authors:** Pablo Urrutia, María F. Díaz, Rodrigo Fernández, Carlos Parra, Lorenzo Tavelli, Leonardo Díaz

**Affiliations:** ^1^Postgraduate Implant Dentistry Department, School of Dentistry, Andrés Bello University, Santiago, Chile; ^2^Perioplastic Institute, Santiago, Chile; ^3^Private Practice, San Fernando, Chile; ^4^Center for Clinical Research and Evidence Synthesis in Oral Tissue Regeneration (CRITERION), Boston, Massachusetts, USA; ^5^Division of Periodontology, Department of Oral Medicine, Infection, and Immunity, Harvard School of Dental Medicine, Boston, Massachusetts, USA; ^6^Department of Prosthodontics, School of Dentistry, University of Chile, Santiago, Chile

**Keywords:** alveolar ridge augmentation, case report, connective tissue, dental esthetics, dental implants

## Abstract

Managing complications related to dental implants placed in the esthetic zone is difficult, often requiring interdisciplinary management to resolve them. This case report is aimed at describing the therapeutic stage approach used to manage complications in an upper central incisor with an implant-supported crown with esthetic, prosthetic, and biological failures. A female patient, 38 years old, consulted for an esthetic deficiency in relation to a crown supported by an implant placed and rehabilitated 8 years ago. The patient showed a high smile line and unfavorable mucosal zenith position associated with the excessive depth and buccopalatal angulation of the implant. Implant removal was performed, followed by vertical and horizontal bone augmentation and soft tissue management in both surgical stages. Then, an implant and a temporary crown were installed in the second-stage surgery to develop the emergence profile of the definitive crown. The 12-month CBCT showed the bone graft well incorporated over time, with increased ridge width and height. In addition, clinical soft tissue examination reported stable soft tissue health in quality and quantity. Management of biological, prosthetic, and esthetic complications in implant dentistry involves a comprehensive knowledge of techniques and disciplines to achieve optimal hard and soft tissue restitution. Proper planning should include an interdisciplinary approach, prosthetic-guided implant placement, stage approach management, and adequate prosthetic rehabilitation.

## 1. Introduction

Survival refers to the presence of the implant and prosthesis at the follow-up examination without specifying their condition. However, success also depends on functional and esthetic aspects, stability of peri-implant tissues, expectations, and patient-reported outcomes [1]. Complications in the esthetic zone are usually clinical challenges requiring interdisciplinary management to solve biological and prosthetic conditions that lead to significant esthetic defects.

While early implant failures are associated with a lack of osseointegration, late implant failures occur when osseointegration has been achieved or the implants are in function [2]. The position of the implant is often considered the most critical factor in determining its removal in cases that do not allow a correct prosthetic restoration from an esthetic and/or functional point of view [3–5]. Other indications for implant removal are related to implants with advanced peri-implantitis [6] or in patients without adequate professional maintenance or with poor oral hygiene [7]. This case report is aimed at describing the therapeutic stage approach used to manage complications in an upper central incisor with an implant-supported rehabilitation with esthetic, prosthetic, and biological failures.

## 2. Case Presentation

### 2.1. Clinical Presentation

**Figure 1:**
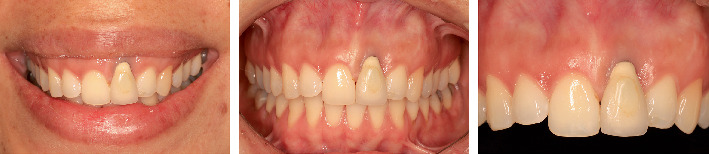
Baseline clinical assessment. The patient had a high smile line and an unfavorable mucosal zenith position related to an implant-supported crown in Zone #21, as well as a shadow due to the lack of buccal volume of the peri-implant tissues.

**Figure 2:**
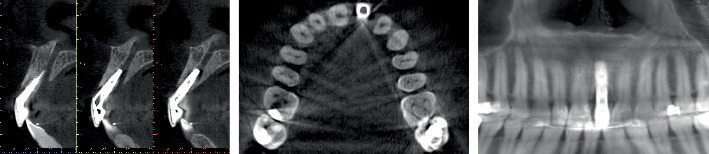
Baseline radiological assessment. Tangential, axial, and panoramic CBCT images of the site. The implant was placed in Zone #21 with excessive depth and buccal–palatal angulation, in addition to the apex projected in the nasopalatine canal, compromising the buccal and palatal plates.

This case report followed the CARE guidelines [8]. A 38-year-old systemically healthy, nonsmoking female patient with no known allergies or ongoing medication use consulted for an esthetic defect in relation to an implant-supported crown rehabilitated 8 years ago. During clinical examination, a screw-retained metal-ceramic prosthesis in Position #21 was observed. The patient showed a high smile line and an unfavorable mucosal zenith position. Due to the lack of soft tissue volume and inadequate attached mucosa (≤ 1 mm), the metal abutment was visible through the mucosa (Figure 1). Preoperative CBCT (ProMax 3D; Planmeca, Helsinki, Finland) revealed an implant placed with excessive depth and buccal–palatal angulation, compromising buccal and palatal plates, and the apex projected into the nasopalatine canal (Figure 2).

### 2.2. Treatment Planning

**Figure 3:**
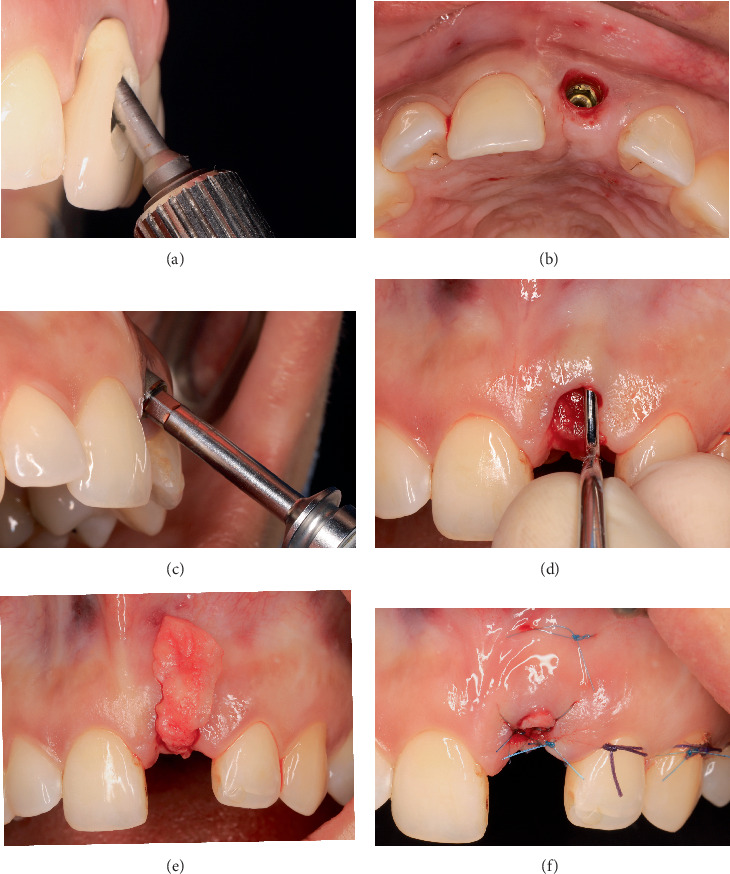
Explantation and soft tissue management. (a, b) Crown removal. (c) Implant removal. (d–f) Rotated pedicle palatal connective tissue flap and vestibular split-thickness tunnel.

An esthetic analysis and patient interview were performed, and the patient reported her dissatisfaction with the esthetic result obtained from the previous treatment regarding the appearance of the prosthesis and her gingiva during a smile. After the treatment objectives were explained, the patient signed an informed consent. To improve local conditions, the crown was removed (Figure 3a). According to Zucchelli et al. [5], due to the more apical position of the mucosal margin in relation to the homologous natural tooth, the facial position of the crown, as well as the implant platform, this peri-implant soft tissue deficiency (PSTD) was considered Class IV-A. Subclass A refers to the tip of both papillae ≥ 3 mm coronal to the mucosal margin of the crown (Figure 3b). Severe implant malposition combined with a thin peri-implant phenotype led to esthetic complications that were impossible to manage predictably, both surgically and prosthetically. Therefore, implant removal was indicated.

### 2.3. Implant Removal and Soft Tissue Management

The implant was extracted with a removal kit (Neo Fixture Remover Kit; Neobiotech, Seoul, Republic of Korea) (Figure 3c). Later, a rotated pedicle palatal connective tissue flap technique was used, as proposed by Scharf and Tarnow [9], performing a vestibular split-thickness tunnel and then suturing with Polyamide 5/0 suture, increasing soft tissue thickness and keratinized mucosa width on the occlusal and buccal aspects of the site (Figures 3d, 3e, and 3f). The donor site was closed with Polyglactin 910 5/0 suture. Subsequently, the healing process was followed periodically for 21 days.

### 2.4. Vertical Bone Augmentation

**Figure 4:**
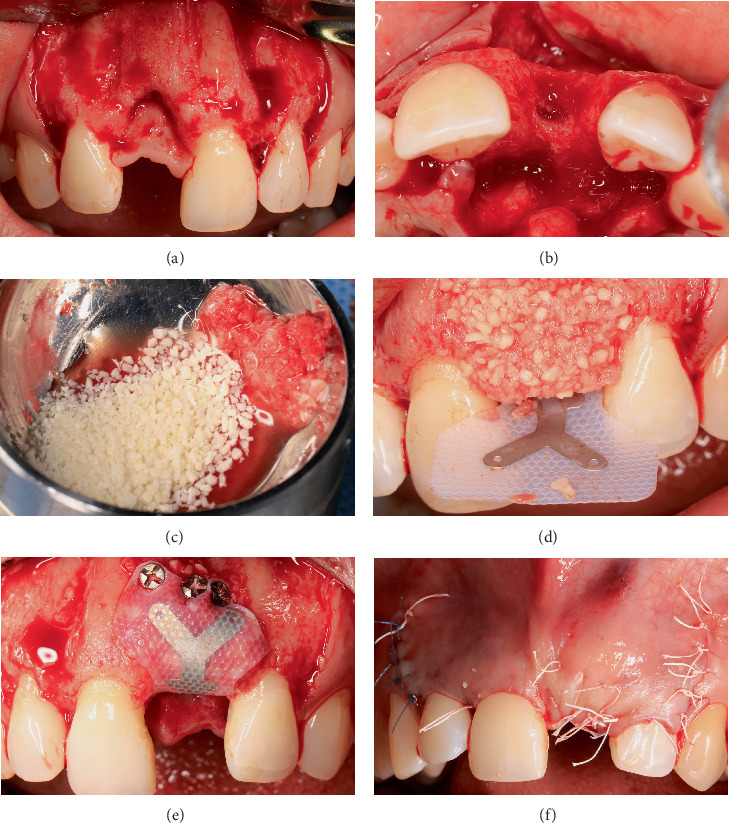
Vertical bone augmentation. (a, b) Frontal and occlusal views of the bone defect. (c) Mixture of autograft with allograft. (d, e) Use of titanium-reinforced PTFE membrane and fixation screws to contain graft mixture. (f) Frontal view of tension-free flap closure.

After 8 weeks, local soft tissue conditions improved, allowing a wide flap design, with vertical release incisions distal to Teeth #13 and #22. The bone defect was evaluated in situ, represented by 9-mm horizontal and 5-mm vertical components. To solve this complex noncontained defect, a titanium-reinforced dense polytetrafluoroethylene (d-PTFE) membrane (Medipac, Stavrochori-Kilkis, Greece) was stabilized with 1.5 × 3.0 mm fixation screws (Pro-fix Precision Fixation System; Osteogenics Biomedical, Texas, United States): two on the palatal side and three on the buccal aspect. The space was filled with a 1:1 mixture of autogenous bone obtained from the same surgical site and particulate mineralized allograft (OraGraft; LifeNet Health, Virginia Beach, Virginia, United States). The previously released flap was closed tension-free with a PTFE 4/0 suture (Figure 4).

### 2.5. Implant Placement and Prosthetic Soft Tissue Management

**Figure 5:**
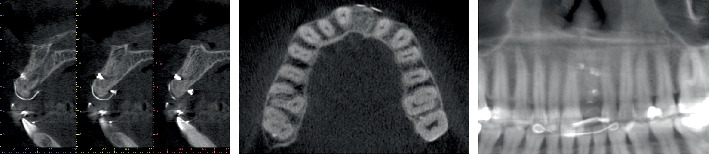
CBCT control at 9 months after bone augmentation. Tangential, axial, and panoramic CBCT images of the site.

**Figure 6:**
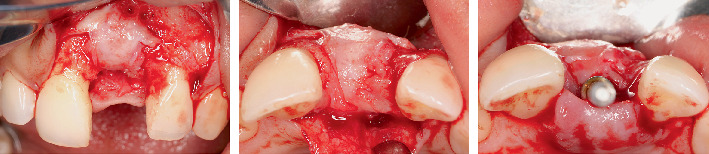
Nine months after bone augmentation. The integration of the graft in the vertical and horizontal dimensions is observed, allowing the placement of an implant in the ideal prosthetic position.

**Figure 7:**
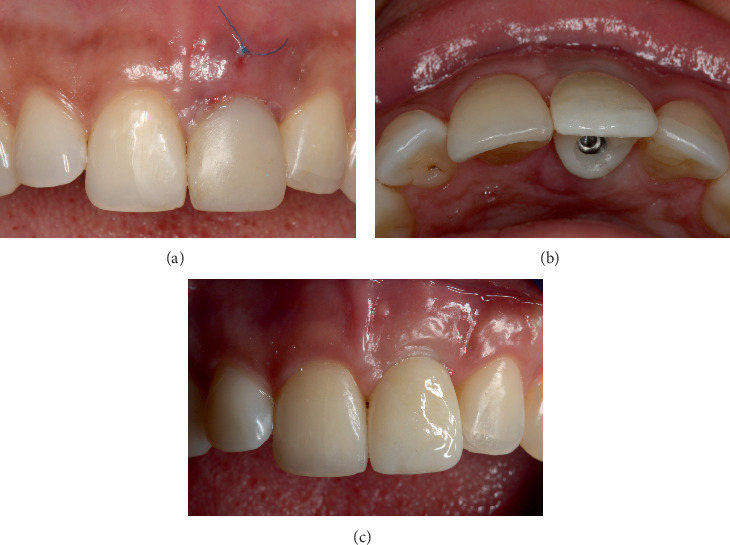
Prosthetic management. (a) Soft tissue augmentation and provisional restoration connected to the implant during the second-stage surgery. (b, c) Definitive lithium disilicate crown in occlusal and frontal views.

A CBCT scan was performed 9 months later to assess graft integration (Figure 5). A flap was opened to remove the membrane and screws, and then a 3.5 × 10 mm implant (IS-III; Neobiotech, Seoul, Republic of Korea) was placed (Figure 6). It was closed with a Polyamide 5/0 suture and waited 4 months. During the second-stage implant surgery, connective tissue grafting was again performed, followed by the delivery of a screw-retained temporary prosthesis (PMMA) to develop the emergence profile and improve the stability of peri-implant tissues over time (Figure 7a). Due to the correct angulation and prosthetic position, it was possible to fabricate a lithium disilicate screw–cement-retained single crown, cemented extraorally to a titanium-base abutment and screwed directly to the implant, avoiding the projection of cement to the peri-implant tissues as a possible source of subsequent infections [10]. Furthermore, it offers excellent versatility in critical and subcritical emergence profile management [11], according to the position and stability of tissues obtained by provisional restoration (Figure 7b,c).

### 2.6. One-Year Follow-Up

**Figure 8:**
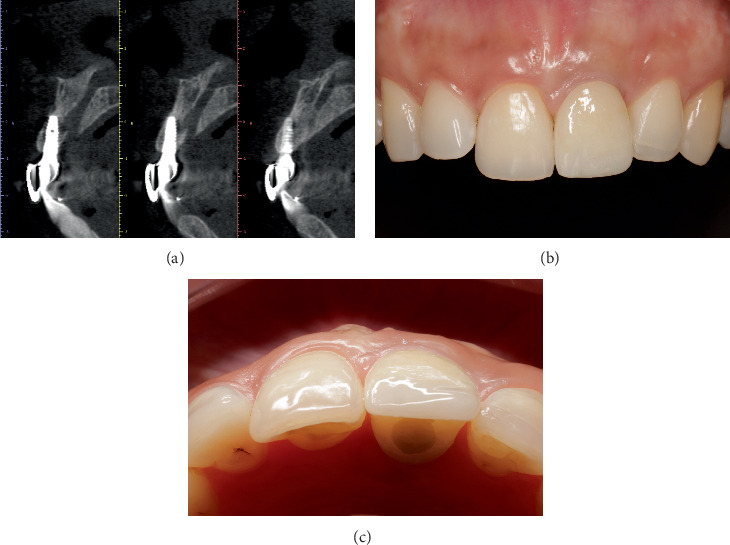
One-year follow-up. (a) CBCT control. (b, c) Frontal and occlusal views. Clinically, the recovery in volume and height of the peri-implant tissues associated with the prosthesis is observed.

The 12-month CBCT showed the bone graft well incorporated over time, with increased ridge width and height (Figure 8a). In addition, clinical soft tissue examination reported stable soft tissue health, both in quality and quantity (Figure 8b,c).

## 3. Discussion

Esthetic, prosthetic, and biological complications associated with implant-supported restorations in the anterior maxilla represent a significant clinical challenge in contemporary implant dentistry. These complications are not only prevalent but are also increasingly emphasized due to heightened patient expectations regarding minimally invasive management and optimal esthetic outcomes. In such cases, clinicians are often confronted with the complex decision of whether to pursue corrective therapies or to remove osseointegrated implants that, despite their functional integration, exhibit unfavorable positioning or soft tissue deficiencies that compromise the final result.

Several factors have been reported that may influence the position and height of the peri-implant mucosal margin [12]. Particularly, the buccal position of the implant can cause the loss of the buccal plate and subsequent mucosal dehiscence [13]. In addition, due to the abutment material and thin peri-implant phenotype, evident discoloration or shadowing of the mucosa was observed [14]. Esthetic complications in the anterior maxilla often result from a combination of soft tissue deficiencies and incorrect three-dimensional implant positioning. Currently, there is evidence of the resolution of this type of complication with soft tissue management and prosthetic replacement, but these protocols are effective when positional failures are moderate [3–5]. According to Mesquita de Carvalho et al. [4], a structured clinical decision-making process based on implant position, hard tissue anatomy, and soft tissue volume is crucial for selecting the appropriate intervention—whether corrective or involving implant removal. In contrast, in cases where implants are positioned buccally or too apically and the esthetic deficit involves both soft and hard tissues, as in PSTD Class IV defects described by Zucchelli et al. [5], the removal of the implant may be the only predictable option. The advantages and disadvantages of implant removal should be considered in terms of explantation techniques, papilla conservation, biological times, morbidity, and patient expectations [15].

Managing esthetic failures of osseointegrated implants requires complex clinical judgment. As highlighted by Monje and Nart [16], malpositioned implants—despite being osseointegrated—may be unmanageable from an esthetic perspective, particularly when placed outside the bony envelope or in patients with high smile lines. Moreover, removal allows for the regeneration of lost hard and soft tissues, which may be necessary to restore esthetic harmony. Implant removal may be questionable due to the increased treatment time, but in this case, it improved the inadequate clinical conditions that caused the malpositioned implant. Therefore, the surgical site without the implant can be considered a bone defect, having enough scientific evidence with predictable protocols such as vertical bone augmentation procedures [17, 18] and the related soft tissue management to generate peri-implant mucosa in quantity and quality that allows greater stability of the implant treatment over time [19].

This case highlights the critical importance of interdisciplinary planning and therapeutic staging in the successful management of complex implant complications. Clinical decisions should be informed not only by implant survival but also by long-term functional stability, esthetic integration, and patient-reported outcomes. The comprehensive treatment in this report exemplifies the synergy between surgical, prosthodontic, and periodontal disciplines—each contributing a distinct yet complementary perspective. Accurate diagnosis and safe explantation required surgical expertise, while prosthodontic planning guided the re-establishment of the emergence profile and ensured a biomimetic restoration. Concurrently, periodontal and peri-implantar surgical techniques enhanced peri-implant tissue volume and architecture, providing the foundation for stable long-term esthetics. This coordinated, stepwise approach underscores the value of interdisciplinary collaboration in the esthetic zone, where clinical demands often exceed the scope of a single specialty. Furthermore, this case reinforces the necessity of individualized treatment planning—tailored to the patient's unique anatomical, biological, and psychological context—rather than relying solely on standardized protocols. As such, interdisciplinary workflows should be regarded as a cornerstone in the management of high-complexity cases involving esthetic, prosthetic, and biological complications.

## 4. Conclusions

Management of biological, prosthetic, and esthetic complications in implant dentistry is highly complex, involving a comprehensive knowledge of techniques and disciplines to achieve optimal hard and soft tissue restitution. Proper planning should include an interdisciplinary approach, prosthetic-guided implant placement, respect for clinical timing, and adequate prosthetic rehabilitation.

## Data Availability

The data that support the findings of this study are available from the corresponding author upon reasonable request.
